# Regional Brain Volume and Thickness in Mild Behavioral Impairment‐Apathy

**DOI:** 10.1002/alz70857_099159

**Published:** 2025-12-24

**Authors:** Daniella Alicia Vellone, Dylan X. Guan, Zahinoor Ismail

**Affiliations:** ^1^ Hotchkiss Brain Institute, University of Calgary, Calgary, AB, Canada

## Abstract

**Background:**

Mild Behavioral Impairment (MBI) is a pre‐dementia syndrome defined by persistent late‐life‐onset behavioural changes and serves as a risk marker for cognitive decline and incident dementia. The apathy MBI domain is hypothesized to be associated with regional brain changes, but it is unclear if these are the same regions described for apathy in dementia. This study investigates volumetric differences between dementia‐free individuals with MBI‐apathy and those without neuropsychiatric symptoms (No NPS).

**Method:**

This study included 446 participants (mean age = 69.6 years; 79.8% cognitively normal; 62.8% female) from the National Alzheimer's Coordinating Center. Participants were grouped into No NPS (*n* = 387) and MBI‐apathy (*n* = 59). Cross‐sectional linear regression models examined the association between NPS status and regional brain measures. Outcomes included AD‐related region composite measures of cortical thickness (entorhinal cortex, inferior temporal gyrus, middle temporal gyrus, inferior parietal lobule, fusiform gyrus, and precuneus) and volume (hippocampus, entorhinal cortex, amygdala, middle temporal gyrus, inferior parietal lobule, and precuneus). Additionally, cortical thickness and volume measures for four apathy‐related regions (anterior cingulate cortex [ACC], orbitofrontal cortex [OFC], dorsolateral prefrontal cortex [dlPFC], and ventrolateral prefrontal cortex [vlPFC]) were included. Models adjusted for age, sex, education, APOE4 carrier status, and Mini‐Mental State Examination score.

**Result:**

Compared to the No NPS group, MBI‐apathy status was significantly associated with lower average AD composite thickness score (β = ‐0.40; 95% CI = ‐0.74‐(‐0.05); *p* = 0.024), total AD composite volume (β = ‐3.33; 95% CI = ‐4.66‐(‐2.00); *p* < 0.001), dlPFC thickness (β = ‐0.35; 95% CI = ‐0.69‐(‐0.004); *p* = 0.048), OFC volume (β = ‐0.34; 95% CI = ‐0.64‐(‐0.04); *p* = 0.028), and dlPFC volume (β = ‐0.27; 95% CI = ‐0.54‐(‐0.01); *p* = 0.040).

**Conclusion:**

MBI‐apathy shows stronger stronger associations with atrophy in regions affected early in the course of AD. This finding suggests that at the CN and MCI stages, MBI‐apathy may reflect brain changes characteristic of early AD. These findings suggest that MBI‐apathy is associated with early‐stage AD and that regions typically linked to apathy are likely affected later in the disease, with stronger associations emerging as AD progresses.

